# Heterogeneity of the Abnormal Prion Protein (PrP^Sc^) of the Chandler Scrapie Strain

**DOI:** 10.3390/pathogens2010092

**Published:** 2013-02-18

**Authors:** Kazuo Kasai, Yoshifumi Iwamaru, Kentaro Masujin, Morikazu Imamura, Shirou Mohri, Takashi Yokoyama

**Affiliations:** Prion Disease Research Center, National Institute of Animal Health, Tsukuba, Ibaraki 305-0856, Japan; E-Mails: kakasai@affrc.go.jp (K.K.); gan@affrc.go.jp (Y.I.); masujin@affrc.go.jp (K.M.); imamuram@affrc.go.jp (M.I.); shirou@affrc.go.jp (S.M.)

**Keywords:** prion, Chandler, small PrP^Sc^ aggregate, conformational stability, PK sensitivity

## Abstract

The pathological prion protein, PrP^Sc^, displays various sizes of aggregates. In this study, we investigated the conformation, aggregation stability and proteinase K (PK)-sensitivity of small and large PrP^Sc^ aggregates of mouse-adapted prion strains. We showed that small PrP^Sc^ aggregates, previously thought to be PK-sensitive, are resistant to PK digestion. Furthermore, we showed that small PrP^Sc^ aggregates of the Chandler scrapie strain have greater resistance to PK digestion and aggregation-denaturation than large PrP^Sc^ aggregates of this strain. We conclude that this strain consists of heterogeneous PrP^Sc^.

## 1. Introduction

Prion diseases, such as scrapie in sheep and goats, bovine spongiform encephalopathy (BSE) in cattle, and Creutzfeldt-Jakob disease (CJD) in humans, are transmissible neurodegenerative disorders [1]. They are characterized by the accumulation of pathogenic isoforms of prion protein (PrP^Sc^), which is a major component of the infectious agent—the prion [1]. PrP^Sc^ is generated by posttranslational modification of the cellular prion protein (PrP^C^). Although PrP^C^ and PrP^Sc^ have identical amino acid sequences, they have different structural and biochemical properties. PrP^Sc^ is defined as an aggregated prion protein (PrP) that is insoluble in detergents and is partially resistant to proteolysis [2,3,4]. Distinct prion entities, referred to as strains, exhibit distinguishable phenotypic traits, including varying incubation periods and lesion profiles, that are heritable in inbred mice [5]. Some strains differ in their PrP^Sc^ properties, e.g., the electrophoretic mobilities associated with different cleavage sites of protease digestion [6], relative glycoform ratios [7] and immunoreactivities against conformation-specific antibodies [8,9]. These findings indicate that prion strain characteristics might be encoded in the structure and/or conformation of PrP^Sc^.

PrP^Sc^ consists of PrPs with various aggregate sizes [10,11,12]. They have been classified based on their density and/or size, as determined by velocity sedimentation in sucrose gradients [12] and flow field-flow fractionation [11]. It has been demonstrated that the most infectious prion entities are present in small aggregates and that prion infectivity is independent of the amount of PrP^Sc^ [11]. According to the seeded aggregation model, the efficiency of PrP^Sc^ conversion depends on the number of active sites located at the tip of growing fibrils [13], and this may explain the higher infectivity of small PrP^Sc^ aggregates. Furthermore, the relationship between infectivity and aggregate size is strikingly different among different prion strains [10]. These results indicate that PrP^Sc^ aggregates of different prion strains have different biochemical features. 

The structural differences in PrP^Sc^ in different prion strains have been analyzed by the conformational stability assay with guanidine hydrochloride (GdnHCl) [14], the aggregation stability assay with sodium dodecyl sulfate (SDS) [15] and the proteinase K (PK)-sensitivity assay [6,16]. The conformational stability of PrP^Sc^, as measured by PrP^Sc^ stability against GdnHCl, has been found to be associated with the incubation period of prion strains [17,18,19]. SDS breaks down large PrP^Sc^ aggregates into smaller particles; thus, the SDS assay measures the aggregation stability of PrP^Sc^ [11], which has been found to be associated with prion replication *in vitro* and *in vivo* [15]. The differing biochemical characteristics of PrP^Sc^ from different prion strains may be related to its structural diversity and may be associated with the biological characteristics of the prion. However, little is known about the correlation between the diversity of PrP^Sc^ aggregates and their biological characteristics, and these are thought to be linked to differences in prion strains. Therefore, a detailed analysis of the biochemical characteristics of PrP^Sc^ based on the aggregation size is necessary to clarify this issue. In this study, the PrP^Sc^ from five mouse-adapted prion strains was classified into small and large aggregates by velocity sedimentation in sucrose gradients. We discovered that small PrP^Sc^ aggregates of the Chandler scrapie strain have characteristics distinct from those of other prion strains.

## 2. Results and Discussion

### 2.1. Size Distribution of PrP^Sc^ Aggregates

**Figure 1:**
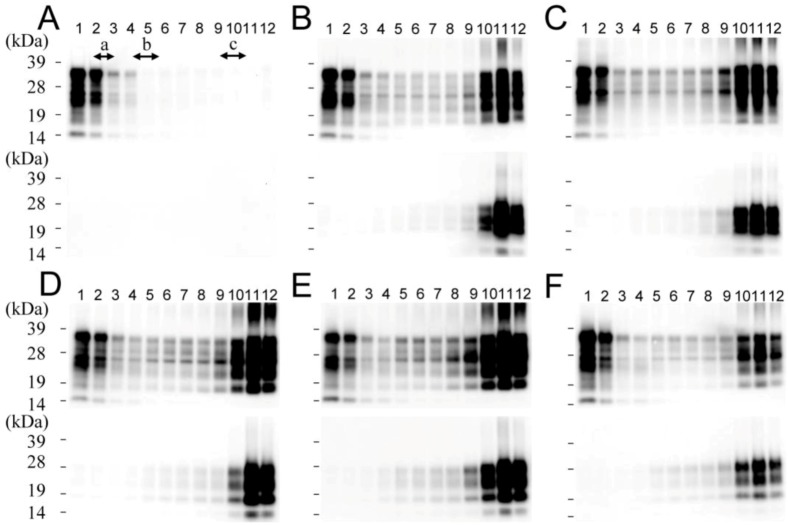
Size distribution of PrP^Sc^ in sucrose density gradients. The brain homogenates of uninfected control mice (**A**) or mice infected with Chandler (**B**), mBSE (**C**), 22L (**D**), ME7 (**E**) or Tsukuba-2 (**F**) strains were fractionated on 10–60% sucrose step gradients. The PrP contents of the fractions were analyzed by Western blotting (upper panels). The fractions shown in the lower panels were treated with PK (50 μg/mL, 37 °C, 30 min) before Western blotting. The molecular mass markers used for size fractionation are a: aldolase (158 kDa), b: thyroglobulin (669 kDa) and c: dextran blue 2,000 (2,000 kDa). The molecular markers for electrophoresis are 39, 28, 19 and 14 kDa.

**Figure S1:**
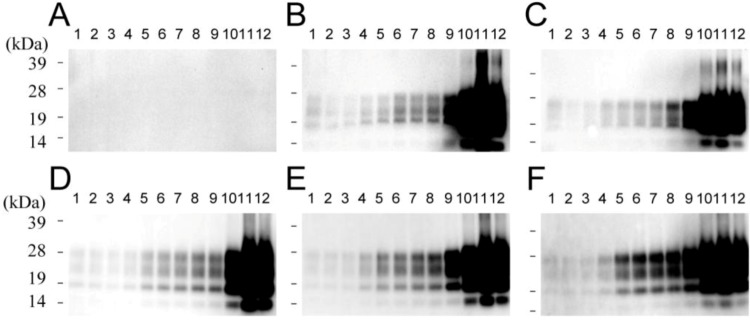
Long-exposure Western blots of PrP^Sc^ in control mice (**A**) and mice affected by the Chandler (**B**), mBSE (**C**), 22L (**D**), ME7 (**E**) and Tsukuba-2 (**F**) strains. The same membranes shown in the lower panel of Figure 1 were exposed for a longer time to visualize the faint PrP^Sc^ signals. The PrP^Sc^ signal was observed in fractions 1–12 for all the prion strains examined. The molecular markers indicated are 39, 28, 19 and 14 kDa.

**Figure S2:**
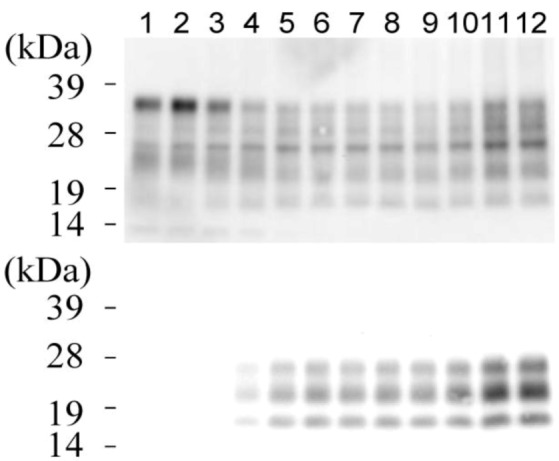
Brain homogenates of Chandler-affected mice were fractionated, and their PrP contents were analyzed by Western blotting. The total amounts of PrP in all fractions were normalized (upper panel), and then each fraction was treated with 4 μg/mL of PK at 37 °C for 30 min. After PK digestion, PrP^Sc^ was found in fractions 4–12 (lower panel). The molecular markers indicated are 39, 28, 19 and 14 kDa.

Using Western blotting, strong PrP signals were observed from fractions 1 and 2 from uninfected mice, and a faint signal was observed from fractions 3 and 4. These signals disappeared upon PK digestion (Figure 1A). Therefore, they were considered PrP^C^ signals. In prion-infected mice, the PrP signal was detected in all fractions (Figure 1B–F). After PK digestion, a strong PK-resistant PrP signal, which was thought to be a PrP^Sc^ signal, was detected in fractions 10–12; a faint PrP signal was also observed in fractions 1–9 for all the prion strains examined (Figure 1 and Figure S1). Upon the digestion of equal amounts of PrP with PK, the PrP signal disappeared from fractions 1–3, but remained distinct in fractions 4–12 (Figure S2), indicating that fractions 1–3 and 4–12 contained mainly PrP^C^ and PrP^Sc^, respectively. PrP fractions were further classified into two groups—fractions 4–9, with small PrP^Sc^ aggregates, and fractions 10–12, with large PrP^Sc^ aggregates—and these fractions were used for the following experiments.

### 2.2. Conformational Stability of Small and Large PrP^Sc^ Aggregates against GdnHCl

**Figure 2:**
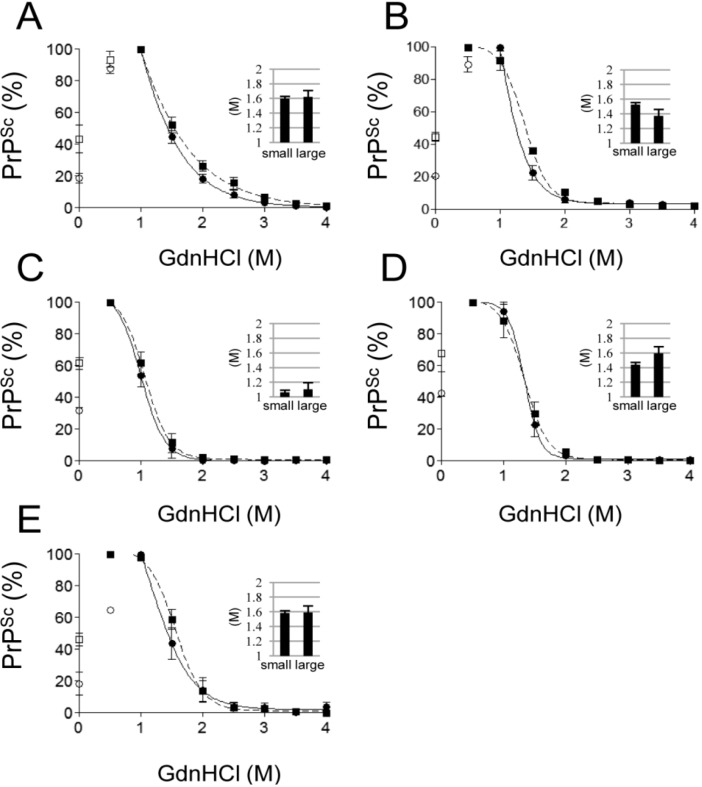
Conformational stability of small and large PrP^Sc^ aggregates against GdnHCl. Small (circle and solid line) and large (square and dashed line) PrP^Sc^ aggregates were treated with different concentrations of GdnHCl. The signal intensity of PrP^Sc^ in Western blots was normalized by using the highest signal intensity observed in each strain (0.5 or 1 M of GdnHCl). Dose-response curves of the Chandler (**A**), mBSE (**B**), 22L (**C**), ME7 (**D**) and Tsukuba-2 (E) strains are shown. Closed circles and squares were used to obtain the curves. Open circles and squares were omitted from the calculation. The inset graphs indicate the concentration of GdnHCl that denatured 50% of PrP^Sc^ ([GdnHCl]_1/2_). Data represent the mean ± SEM of four independent experiments.

The conformational stability of PrP^Sc^ aggregates was assessed by GdnHCl treatment. The total amounts of PrP^Sc^ in small and large PrP^Sc^ aggregate fractions were normalized and then assayed. Both fractions showed a similar bimodal behavior. After treatment with low concentrations of GdnHCl, the detectable amount of PrP^Sc^ was increased, whereas it was reduced by higher concentrations of GdnHCl. This increased immunoreactivity at low concentrations of GdnHCl (0.5 or 1 M), compared with the immunoreactivity without GdnHCl treatment, might be explained by the characteristics of mAb T2, which recognizes a discontinuous epitope [35]. The detectable PrP^Sc^ was thought to be a non-solubilized precipitant. No difference was observed in the reduction curves or [GdnHCl]_1/2_ values between small and large PrP^Sc^ aggregates in all the prion strains examined (Figure 2). 

### 2.3. Aggregation Stability of Small and Large PrP^Sc^ Aggregates against SDS

**Figure 3:**
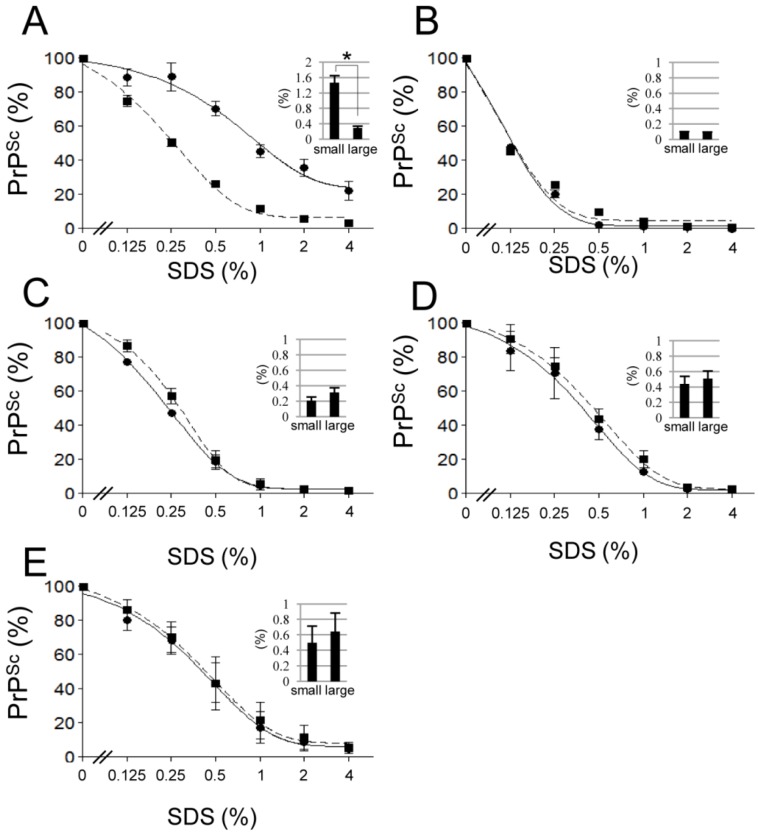
Aggregation stability of small and large PrP^Sc^ aggregates against SDS denaturation. Small (circle and solid line) and large (square and dashed line) PrP^Sc^ aggregates were treated with different concentrations of SDS. The PrP^Sc^ signal intensity of each sample was compared to that of the non-treated (0% SDS) sample. Dose-response curves for the Chandler (**A**), mBSE (**B**), 22L (**C**), ME7 (**D**) and Tsukuba-2 (**E**) strains are plotted. The inset graphs indicate the concentration of SDS that denatured 50% of PrP^Sc^ ([SDS]_1/2_). Data represent the mean ± SEM of four independent experiments. The statistical significance of the difference between the [SDS]_1/2_ of small and large PrP^Sc^ aggregates was assessed by Student’s *t*-test (*p < 0.001).

The aggregation stability of small and large PrP^Sc^ aggregates was assessed by SDS denaturation. The dose response was strikingly different between small and large PrP^Sc^ aggregate fractions in the Chandler strain (Figure 3A). Small PrP^Sc^ aggregates had significantly higher [SDS]_1/2_ than the large aggregates (inset graph in Figure 3A). However, no significant difference was observed between the [SDS]_1/2_ for small and large PrP^Sc^ aggregates in other prion strains (Figure 3, B–E).

### 2.4. Comparison of the PK Sensitivity of Small and Large PrP^Sc^ Aggregates

**Figure 4:**
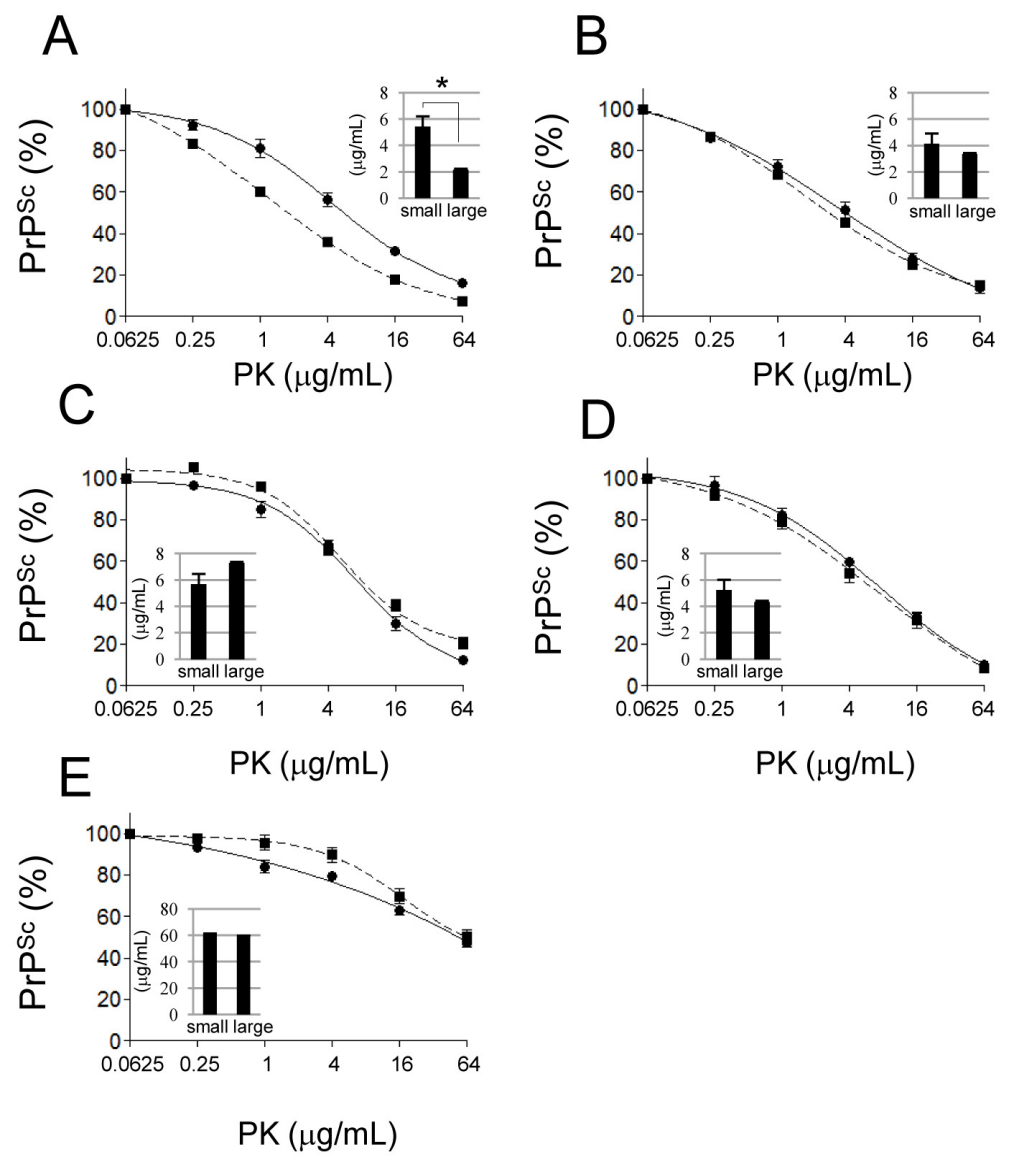
Comparison of the proteinase K (PK)-sensitivity of small and large PrP^Sc^ aggregates. Small (circle and solid line) and large (square and dashed line) PrP^Sc^ aggregates were treated with different concentrations of PK. The PrP^Sc^ signal intensity of each sample was compared to that of the 0.0625 μg/mL PK-treated sample, which showed the highest signal intensity. Dose-response curves from Chandler (**A**), mBSE (**B**), 22L (**C**), ME7 (**D**) and Tsukuba-2 (**E**) strains have been provided. The inset graphs indicate the concentration of PK that digested 50% of PrP^Sc^ ([PK]_1/2_). PrP^Sc^ was detected by mAb T2. Data represent the mean ± SEM of four independent experiments. The statistical significance of the difference in the [PK]_1/2_ of small and large PrP^Sc^ aggregates was assessed by Student’s *t*-test (*p < 0.01).

No significant differences were found in the [PK]_1/2_ values or dose-response reduction curves of small and large PrP^Sc^ aggregates in the mBSE, 22L, ME7, and Tsukuba-2 strains (Figure 4, B–E). However, small PrP^Sc^ aggregates of the Chandler strain showed greater PK resistance than large aggregates of the same strain (Figure 4A). 

### 2.5. Transmissibility of Small and Large PrP^Sc^ Aggregates in the Chandler Strain

Since the small and large PrP^Sc^ aggregates of the Chandler scrapie strain exhibited different biochemical features, we examined the transmissibility of each fraction. The incubation periods of fractions 5, 7, 9 and 11 were the same in the Chandler strain (Table 1). In contrast, the incubation periods of mBSE fractions differed: fractions 5 and 7 showed significantly longer incubation periods than fractions 9 and 11 (Table 1). The PrP signal intensities of fractions 5 and 7 were lower than those of fractions 9 and 11 in both strains. These results indicate that the transmissibility of the Chandler strain was independent of PrP signal intensity, whereas that of the mBSE prion strain was dependent upon PrP signal intensity.

**Table 1:** Incubation period of fraction samples

Fraction No.	Chandler				mBSE		
	Incubation period ^1^	n/n_0_^2^	PrP ^3^		Incubation period	n/n_0_	PrP
5	176.5 ± 4.6	4/4	0.11		241.8 ± 13.0*	4/4	0.16
7	174.5 ± 0.3	4/4	0.10		222.8 ± 6.3*	4/4	0.19
9	178.5 ± 1.8	4/4	0.26		216.0 ± 12.7	4/4	0.54
11	175.3 ± 0.6	4/4	1.00		196.0 ± 12.7	4/4	1.00

^1^ Mean ± SD (days). ^2^ n, number of mice developing clinical signs of prion disease; n_0_, number of mice inoculated. ^3^ PrP content is indicated by the signal intensity ratio relative to that of fraction 11. * Student’s *t*-test comparing the incubation period for each inoculum against that of fraction 11 within each strain (*p* < 0.01).

### 2.6. Discussion

We classified the PrP^Sc^ aggregates of five mouse prion strains based on their density and/or size by using the velocity sedimentation method. There were no significant differences in the PrP distribution patterns among the prion strains tested in this study. This is consistent with results from a previous report [12]. PrP^C^ was found mainly in fractions 1–3, which were the fractions with the lowest density, and PrP^Sc^ was found mainly in fractions 4–12 (Figure 1 and Figure S2). PrP^Sc^ was further classified into two groups: small PrP^Sc^ aggregates (fractions 4–9) and large PrP^Sc^ aggregates (fractions 10–12). The results showed that fractions 4 and 10 contained molecules with a molecular weight of approximately 669 and 2,000 kDa, respectively. Thus, small and large PrP^Sc^ aggregates are estimated to consist of 20–60 and >60 PrP^Sc^ molecules, respectively, if the aggregates are composed entirely of PrP^Sc^ molecules with a molecular weight of 35 kDa. Fractions 4–9 and fractions 10–12 have been defined as PK-sensitive PrP^Sc^ (PrP^Sc^-sen) and PK-resistant PrP^Sc^ (PrP^Sc^-res) aggregates, respectively, in a previous study [12]. However, we demonstrated that small PrP^Sc^ aggregates resisted to PK digestion to the same degree as large PrP^Sc^ aggregates; both PrP^Sc^ was converged to PrP 27-30 after PK digestion (Figure 4 and Figure S2). This difference may be caused by the different amounts of PrP^Sc^ examined in this study and the previous study. The previous study did not use equal amounts of protein from the two fractions. In contrast, we used equal amounts of small and large PrP^Sc^ aggregates for the biochemical assays, thus more clearly measuring their resistance to PK digestion. We also confirmed, by adding excess amounts of bovine serum albumin, that the total protein concentration of the sample did not influence the result (data not shown). Furthermore, the small PrP^Sc^ aggregates in the Sc237 scrapie-affected hamster, which were thought to be PrP^Sc^-sen in the previous study [12], also resisted to PK digestion (data not shown). It has been reported that the small and the large PrP^Sc^ from prion-infected cultured cells resisted to PK digestion [20]. In this study, we also confirmed that the small and the large PrP^Sc^ aggregates showed similar PK-resistance. We conclude that sucrose gradient sedimentation could not discriminate PrP^Sc^-sen from PrP^Sc^-res. Further studies are required to determine the characteristics of PrP^Sc^-sen.

Here, we demonstrated the PrP^Sc^ heterogeneity of the Chandler scrapie strain. Small PrP^Sc^ aggregates were relatively more resistant to PK digestion and SDS denaturation than large PrP^Sc^ aggregates in this strain. This finding suggests that conformationally stable small PrP^Sc^ aggregates and relatively unstable large PrP^Sc^ aggregates co-exist. It has been proposed that prions consist of a variety of PrP^Sc^ species and that the species that replicates most efficiently becomes the predominant species in an environment [21,22,23]. PrP^Sc^ heterogeneity has also been reported in the brains of CJD patients [24] and scrapie sheep [25,26]. The 79A, 139A and Chandler strains were established during the transmission of natural sheep scrapie samples to sheep, goat and mice [27]. Cell culture models also showed that the 139A prion strain consists of both 139A-like and 79A-like prion strains [28]. Thus, the Chandler strain and its derivatives may harbor multiple PrP^Sc^, and our results may reflect this variety. Transmissibility in the fractions was not associated with the amount of PrP^Sc^ in the Chandler strain (Table 1). This finding might also support the heterogeneity of this strain.

Clearer understanding of the structure and/or conformation of PrP^Sc^ is required in order to better understand the relationship between these characteristics and prion transmissibility. We believe that the Chandler strain will be a good model for addressing this question. 

## 3. Experimental Section

### 3.1. Animals and Prions

All the animal experiments were reviewed by the Committee Responsible for Ethics in Animal Experiments at the National Institute of Animal Health. We used mouse scrapie strains (Chandler, 22L, ME7 and Tsukuba-2) and the mouse-adapted classical BSE strain (mBSE) in this study [29,30,31,32]. Mice brains were homogenized in 9 volumes of phosphate-buffered saline (PBS; pH 7.4) at 3,000 rpm for 2 min by using a multi-beads shocker (Yasui-Kikai, Osaka, Japan). After brief centrifugation, 20 μL of the supernatant was intracerebrally inoculated into 3-week-old female ICR mice (SLC, Hamamatsu, Japan). Diseased mice were euthanized and sacrificed, and their brains were collected for PrP^Sc^ examination [33].

### 3.2. Velocity Sedimentation in Sucrose Gradients

Velocity sedimentation of PrP^Sc^ in sucrose gradients was performed as previously described, with minor modifications [12]. Six hundred micrograms of 10% brain homogenate was lysed in 300 μL of TN buffer (10 mM Tris, 150 mM NaCl, pH 7.4) with 2% (v/v) Triton X-100 and 1% (w/v) sodium *N*-lauroyl sarcosinate (Sarkosyl) at 4 °C for 30 min. Insoluble material was removed by a 1-min spin at 17,000 × *g* at 4 °C. Sucrose gradients were formed in polyallomer (13 × 51 mm) tubes with 450 μL of each of the following sucrose concentrations: 10, 15, 20, 25, 30 and 60% in TNS (10 mM Tris, 150 mM NaCl, 1% Sarkosyl, pH 7.4), and the sample was loaded on the top. The gradients were spun for 1.5 h at 4 °C and 50,000 rpm (*g*_av_ = 200,000 × *g*) in an MLS-50 rotor in an Optima MAX-E ultracentrifuge (Beckman Coulter, Fullerton, CA, USA). Twelve fractions (250 μL each) were collected from the top of the tube. Aliquots of the samples were boiled for 5 min in SDS loading buffer and subjected to Western blot analysis [34]. The PrP protein was detected with the anti-PrP monoclonal antibody T2 [35]. The intensities of the PrP signals were compared, and the samples were diluted in TNS to normalize the amount of PrP in each sample. The samples were used for the following experiments.

### 3.3. Conformational Stability Assays

To determine the structural stability of PrP^Sc^ without PK digestion, the conformational stability and solubility assay [36] was employed. For assessing conformational stability, 80 μL of sample was mixed with an equal volume of the following concentrations of GdnHCl: 0, 1, 2, 3, 4, 5, 6, 7 and 8 M. After 1 h treatment at 37 °C, samples were diluted with 1.04 mL TS buffer (100 mM Tris, 2% Sarkosyl, pH 7.4). Subsequently, samples were adjusted to a final concentration of 0.5 M GdnHCl and incubated for 1 h at 37 °C. 

To assess the aggregation stability, 80 μL of sample was mixed with an equal volume of the following concentrations of SDS: 0, 0.25, 0.5, 1, 2, 4 and 8% (w/v). Samples were incubated for 30 min at 70 °C and then diluted with 1.04 mL of TS buffer. 

Non-solubilized PrP^Sc^ was precipitated by centrifugation at 20,000 × *g* for 1 h at 22 °C. The precipitated sample, referred to as GdnHCl- or SDS-resistant PrP^Sc^, was subjected to Western blot analysis. The PrP signal intensities of the experimental samples were normalized to the sample that showed the highest intensity in each experiment: 0.5 or 1 M of GdnHCl or 0% of SDS values. The data were fit into a sigmoidal dose-response curve by using the Graph Pad Prism Software (Graph Pad Prism Software, Inc., San Diego, CA). The half-maximal effective concentrations of GdnHCl ([GdnHCl]_1/2_, M) and SDS ([SDS]_1/2_, %) were determined.

### 3.4. PK-Sensitivity Assay

Fractionated samples were incubated with varying concentrations of PK (final concentrations of 0.0625, 0.25, 1, 4, 16 and 64 μg/mL) at 37 °C for 30 min. Samples were boiled for 5 min in SDS loading buffer and subjected to Western blot analysis, as described above. PrP signals were normalized to the 0.0625 μg/mL PK-treated sample, which showed the highest signal intensity. Data were fit as mentioned above, and the half-maximal effective PK concentration ([PK]_1/2_, μg/mL) was then determined.

### 3.5. Incubation Period Assay

Two hundred microliters of fractionated sample were dialyzed against PBS at 4°C for 2 d to remove detergents. The sample volume was adjusted to 150 μL by using a centrifugal filter device (YM-10; Millipore, Billerica, MA), and then 20 μL of the sample was inoculated intracerebrally into 3-week-old female ICR mice. After inoculation, the clinical status of the mice was monitored daily to assess the onset of neurological signs. Diseased mice were sacrificed and then examined for PrP^Sc^.

## 4. Conclusions

The small PrP^Sc^ aggregate, previously thought to be PrP^Sc^-sen, is resistant to PK digestion. Thus, the size fractionation method cannot discriminate PrP^Sc^-sen from PrP^Sc^-res. Furthermore, the small PrP^Sc^ aggregates of the Chandler strain have greater resistance to PK digestion and aggregation-denaturation than the large PrP^Sc^ aggregates of this strain. These results suggest that this strain consists of heterogeneous PrP^Sc^. Detailed analysis of the biochemical characteristics of PrP^Sc^ on the basis of the aggregation size will determine the heterogeneity of other prions.
